# Emerging Artificial Intelligence Technologies for Evaluation of Dental Composite Restorations: A Scoping Review

**DOI:** 10.1055/s-0046-1816536

**Published:** 2026-02-12

**Authors:** Selçuk Yılmaz, Hatice I. Yılmaz, Negar Jamshidi

**Affiliations:** 1 Independent Researcher, Beverlo, Belgium; 2 Monash Addiction Research Centre, Monash University, Richmond, Victoria, Australia; 3 School of Law, Society and Criminology, University of NSW, Sydney, New South Wales, Australia

**Keywords:** clinical decision-making, convolutional neural networks, deep learning, dentistry, machine learning

## Abstract

Artificial intelligence (AI) is increasingly applied in restorative dentistry, but its role in assessing dental composite restorations is not yet well established. This scoping review evaluated how AI has been used to assess mechanical, esthetic, and diagnostic aspects of composite restorations. A systematic search of PubMed, Embase, Scopus, and Web of Science from 2020 to July 30, 2025, identified studies that applied AI to assess dental composite restorations. Fourteen studies met the inclusion criteria, and all but one had a low to moderate risk of bias. Reported AI applications included tooth shade matching, evaluating mechanical properties, classifying restoration types from images and radiographs, predicting clinical performance, assessing cure depth and microtensile bond strength to estimate debonding risk, and identifying factors associated with marginal microleakage. The highest reported predictive accuracies were achieved by an artificial neural network predicting abrasive wear (99.7% accuracy), adaptive boosting and multilayer Perceptron models predicting flexural strength and Vickers hardness (up to 99.0 and 98.9% accuracy, respectively), and extreme gradient boosting predicting mechanical properties (98.8–99.6% accuracy), each on task- and dataset-specific internal validation. Overall, current AI models show promise for supporting composite restoration evaluation but remain at an early, proof-of-concept stage. The use of small, often single-center samples and the absence of external validation suggest that their performance metrics may be overly optimistic and not yet ready for clinical application. Larger, multi-center datasets, transparent reporting, external validation, and formal assessments of clinical utility are needed before these tools can be considered for routine clinical application.

## Introduction

Artificial intelligence (AI) represents a rapidly advancing area of machine intelligence that seeks to replicate complex human cognitive functions, facilitating machines to perform tasks traditionally associated with human intellect. 1 This involves developing algorithms that enable machines to assimilate information from data and perform fundamental tasks, a process known as Machine Learning. 2 More sophisticated systems, such as Deep Learning, a subset of Machine Learning, leverage neural networks with multiple layers and facilitate the resolution of more complex tasks through feature extraction and pattern recognition. 3 4 This enables deep learning algorithms to frequently outperform other machine learning strategies in identifying patterns across extensive, diverse datasets. 5 Furthermore, this capability is especially beneficial in dentistry, where datasets typically include images, radiographs, and clinical records. 6

In recent years, AI technologies have rapidly progressed from simple Machine Learning models to more complex Deep Learning and Convolutional Neural Networks, greatly enhancing dental imaging analysis and restorative procedures. 7 8 The integration of AI into digital dentistry has further advanced, facilitating personalized and efficient dental care. 9 10 Considering that composite restorations represent a substantial portion of restorative treatments worldwide, with failure rates impacting patient satisfaction and healthcare expenditure, improving their evaluation using AI is of considerable clinical relevance. 11

The current body of research highlights a gap in the thorough application of AI models specifically designed to assess composite restorations, as most research tends to focus more broadly on dental restorations in general or on detecting dental caries. 12 13 14 The lack of standardized protocols and limited clinical validation further impedes the routine use of AI tools, potentially affecting the accuracy of diagnoses and treatment outcomes. 15 16 Therefore, addressing these shortcomings is crucial to leverage the capabilities of AI in restorative dentistry fully and to minimize the risk of misdiagnosis.

The recent emergence of large, expert-annotated datasets of periapical radiographs, such as DenPAR, reflects a growing effort to develop robust benchmarks for dental AI. 17 In parallel, multimodal and multi-center datasets that combine panoramic radiographs, periapical images, and CBCT scans, as well as panoramic benchmarks such as the DENTEX challenge, highlight that effective AI performance often depends on detailed tooth- and restoration-level labeling and images collected from multiple centers and devices. 18 19 Despite these advances, current datasets and benchmarks still focus mainly on anatomical structures, general restorative conditions, and oral lesions, with limited provision of explicit labels for dental composite restorations or detailed data on restorative materials. 17 20 This emphasizes the critical need for datasets and models tailored explicitly to dental composite restorations. 20 Accordingly, this review identifies and evaluates the performance of all published AI models developed for this purpose.

## Methods

We opted to perform a scoping review rather than a systematic review to explore a broader range of questions related to mechanical and esthetic characteristics of dental composite restorations. This proposed review is structured according to the scoping review framework established by Arksey and O'malley 21 and is reported in accordance with the guidelines provided by the Preferred Reporting Items for Systematic Reviews and Meta-Analyses extension for Scoping Reviews (PRISMAScR). 22 The checklist is presented in Supplementary Material (available in the online version only). No formal protocol was prospectively registered for this scoping review; however, the full search strategies, screening forms, and data–extraction templates are provided in Supplementary Material (available in the online version only).

### Search Strategy

An extensive search strategy was designed by integrating both free text and controlled vocabulary terms to address the primary concepts: (1) AI intervention and (2) composite resin restorations ( Supplementary Material , available in the online version only). Initially, this strategy was developed for a PubMed search and later adapted for use in Embase, Scopus, and Web of Science databases. The search was limited to the last 5 years to capture recent advancements. No language limitations were applied to the search strategy. Titles and abstracts in languages other than English were evaluated using a mix of English abstracts and automated Google translation tools. Nevertheless, only studies that allowed for a dependable assessment of methods and outcomes were considered. The complete search strategies for all databases, together with the number of records retrieved from each, are provided in Supplementary Tables S1 to S5 (available in the online version only).

### Eligibility Criteria

The review included studies that examined the use of AI or ML methodologies in the context of dental composite-resin restoration. In addition, the datasets and benchmarks used for comparison (expert opinion or reference standards) with the model had to be clearly identified, and the studies' outcomes needed to be measurable (predictive or quantifiable). Furthermore, experimental, in vitro, or in vivo studies investigating mechanical or aesthetic characteristics of dental composite resin restorations were included. Studies on temporary materials, hybrid ceramics, or dentures were omitted since the review focuses on resin-based composites for permanent restorations. Studies in which restorations were labeled only as “fillings” without specifying the use of resin-based composite were excluded, unless the composite could be clearly separated from other materials in the analysis. Studies that did not report numerical or measurable outcomes on mechanical or aesthetic properties were excluded to allow for quantitative comparisons. Additionally, AI studies not related to dentistry, expert opinions, commentaries, letters to the editor, theses, conference abstracts, and any studies published prior to 2020 were not considered ( Supplementary Table S6 , available in the online version only). To identify additional relevant records, the reference lists of the selected studies and associated systematic reviews were meticulously examined.

### Screening and Data Extraction

Following the search of each database, potential records were deduplicated using EndNote X9.3.3 (Clarivate Analytics, Pennsylvania, United States). The records were independently screened by two reviewers based on the eligibility criteria to ensure accuracy and reduce bias. Full-texts of eligible records were independently reviewed by two reviewers, with any disagreements resolved by consensus with a third reviewer. Initially, data extraction from all eligible records was conducted by one reviewer, with a second reviewer subsequently verifying the data. Standardized screening and data–extraction templates were used for study selection and charting; the full screening and extraction templates are provided in Supplementary Material (available in the online version only). The extracted data included information on study authors, publication date, country, study focus, AI model and approach, sample characteristics (sample size, sample used), composite type, instrument used for analysis, validation type and validation data size, AI model accuracy, and main conclusions. For each included study, a standardized data–extraction template captured dataset size, class balance (where reported), validation strategy (e.g., single hold-out split, k-fold cross-validation), and external/multicenter validation. These characteristics are presented for each study in a summary table in Supplementary Table S1 (available in the online version only). We additionally extracted, for each included AI study, whether an AI-specific reporting guideline (TRIPOD–AI or STARD–AI) was mentioned, whether source code and/or data were publicly available or derived from public datasets, and whether any model calibration or formal clinical utility analysis (e.g., calibration plots, decision–curve analysis) was reported. These items are summarized in Supplementary Table S2 (available in the online version only). Due to significant methodological heterogeneity, a meta-analysis was not feasible.

### Risk of Bias Assessment

Since the included studies varied widely in their broader approach and main objectives, the most appropriate risk-of-bias tool was used to assess each study's quality. For studies primarily on developing and evaluating AI models to predict dental composite performance outcomes, PROBAST (Prediction Model Risk of Bias Assessment Tool), designed for model development and external validation of predictive algorithms, was utilized. 23 In studies with methodologies and foundations based on in vitro experimental data, the QUIN (Quality Assessment Tool for In Vitro Studies) risk-of-bias tool for in vitro dental studies was applied. 24 For studies on diagnostic accuracy of AI models, the QUADAS-2 (Quality Assessment of Diagnostic Accuracy Studies-2) tool was used to assess bias across four domains: patient selection, index test execution, reference standard execution, and flow and timing. 25 The risk of bias assessment was conducted by one reviewer, with a second reviewer verifying the quality assessment. Any disagreements or uncertainties were addressed through discussion until a consensus was reached.

## Results

### Main Characteristics

**Fig. 1:**
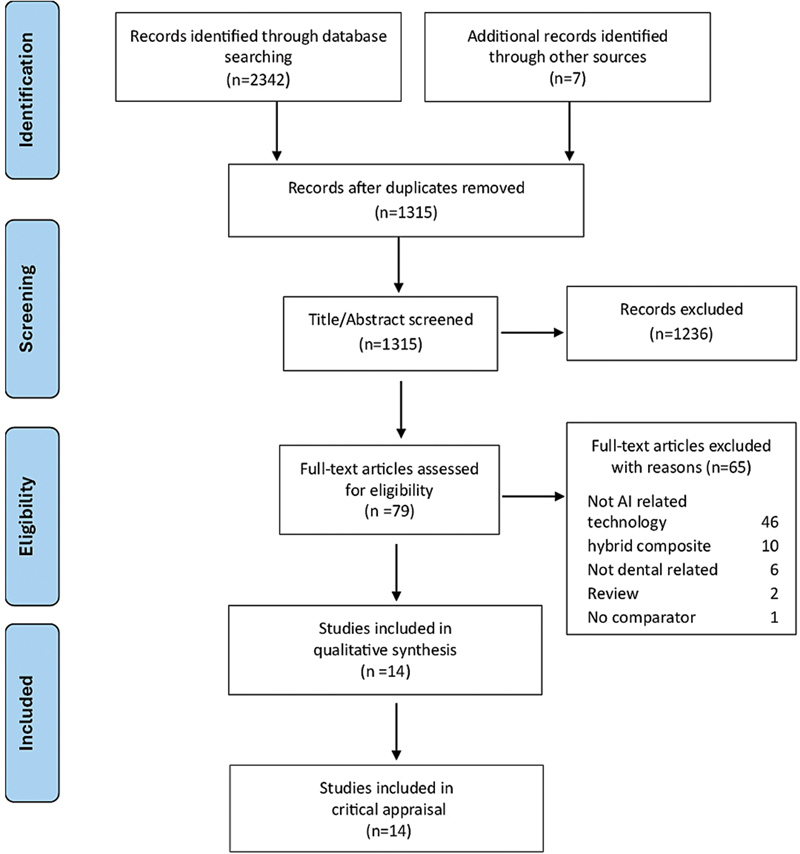
PRISMA flow chart.

The initial search process identified 2,349 potential articles. After removing duplicates, 1,315 articles had their titles and abstracts screened based on predefined eligibility criteria. Subsequently, 1,236 records were found ineligible and excluded, leaving 79 potential records for detailed full-text examination. After reviewing the full texts, 14 studies were deemed eligible for inclusion in the final analysis and critical appraisal ( Fig. 1 ). The primary reasons for exclusion were studies unrelated to AI technology ( *n* = 46), on hybrid composite materials ( *n* = 10), and studies not related to dentistry ( *n* = 6). The key characteristics of the eligible studies are summarized in Table 1 . Over a third of the studies were conducted in India ( *n* = 5), with two studies each in the United States and multiple countries. The remaining studies were conducted in Germany, Iran, Japan, the Philippines, and Turkey, with one study in each country.

**Table 1:** Characteristics of included studies

Author, year, country	Focus of study	AI model/approach	Sample used/composite type (sample size)	Instruments for analysis	Validation type (data size)	AI model accuracy	Conclusion
Almoro et al, 2024, Philippines	Image recognition of shade matching for CRs	SVM DL-based CNN	Images and Tetric N-Ceram Shade Guide (1,253 intraoral images)	Mobile application system	Cross-validation using train -Split test (30% of training set) User testing (20 images)	SVM model: 68.5% Mobile application user: 90%	Mobile application uses DL (CNNs and SVMs) for precise dental shade matching, providing an alternative to visual assessments. With 90% accuracy in User testing, future work includes dataset enrichment and new feature extraction for SVM improvement
Dilian and Kadhim, 2022, Iraq	Compare the microleakage of composite resins	ML (random forest model)	Tetric EvoCeram Bulk Fill 3M Filtek Bulk Fill (60 extracted maxillary premolars prepared with Class II cavity)	Micro-CT for microleakage analysis	Not reported (84 observations and 500 trees)	RF 67.1%	Random Forest model identified composite type as a key factor affecting marginal microleakage. Preheated bulk-fill composite showed less microleakage than bulk-fill flowable composite. This suggests preheating may minimize microleakage
Engels et al, 2022, Germany	Develop an AI system to detect and categorize posterior dental restorations from intraoral photos	DL-based CNN (transfer learning: ResNeXt-101–32 × 8d)	Images in six categories: 483 Unrestored teeth 570 composite 213 Cement 278 Amalgam 125 Gold 92 Ceramic (1,761 intraoral images)	Computational tools and software: Python: v3.8 PyTorch library v1.8.1 Torchvision: v0.9.1	Single train-test split (20% of the dataset) Expert evaluation (reference standard)	Overall > 90% Unrestored teeth 94.9% Composites 92.9% Amalgam 99.2% Gold 99.4% Ceramic: 97.8%	The CNN accurately classified posterior restorations in intraoral photographs, achieving at least 90% diagnostic accuracy for dental restorations
Karatas et al, 2021, Turkey	Evaluate the CNN's effectiveness in identifying dental restorations from bitewing and periapical radiographs	DL-based CNN (transfer learning: Resnet34 architecture)	Bitewing and Periapical radiographs (total: 500, 275 bitewings, 275 periapical images)	PSP Scanning Machine: KaVo Scan eXam One Intraoral X-ray Machine: KaVo FOCUS Digital radiographic images were retrieved from PACS (picture archiving and communication system) DL models used FastAI library and PyTorch backend	5-fold cross-validation Single train-test split (20% of the dataset)	Overall > 90% Amalgam 88% Composite resin 87% Metal-ceramic 96%	DL-based CNNs applied to dental radiographs offer a promising method for identifying dental restorations such as amalgam, composite resin, and metal-ceramic The model has achieved clinically acceptable success in recognising these restoration types
Paniagua et al, 2025, United States	Efficacy of AI and ML to predict performance outcomes of dental composites based on their composite attributes	ML classification algorithms (9 models) a Regression analysis (5 models) a	Commercial composite samples (233)	Model performance assessment: to assess the performance of ML models Classification algorithms and regression models	Single train-test split (20% of data)	KNN 90% Classifications: FlexStr decision tree, random forest, and XGBoost 73% ShrinkV Decision tree 81% Random forest 91% XGBoost 89% ShrinkStr Logistic regression, SVM, and XGBoost 89% Logistic regressions 96%	The study developed a crucial dataset and demonstrated AI's potential in dental material science, while identifying the need for comprehensive data to optimize dental composite design and material development
Rocha et al, 2022, United States, Canada, Brazil	Evaluate the cure depth of resin-based composites (RBCs)	Artificial neural network (ANN)	RBCs (150) conventional RBCs Admira Fusion, Estelite Σ Quick, Filtek Supreme Ultra, Herculite Ultra, Mosaic, and Tetric Evoceram Bulk-filled RBCs SureFil SDR flow + , Tetric Powerflow X-tra Fi	Integrating sphere Fiber-optic spectrometer Digital caliper Laser beam analyzer Machine learning software: SAS Viya	Not reported	Alpha standard error (ASE) of 0.004	Dental LCUs may not achieve sufficient cure depth with brief exposures. Effective curing depends primarily on radiant exposure in blue and total spectral ranges, wavelengths, and adequate exposure time, rather than irradiance alone
Shubham and Banerjee, 2024, India	Evaluate an ML model to predict dental restoration, aiming for high accuracy	Hybrid CNN and KNN	Dental images (10,220 photos: 3,360 Amalgam 3,450 Composite 3,410 Metal-Ceramic)	Primarily used CNN and KNN models for analysis	Single train-test split (not reported)	Overall: 98.2% 98.2% Amalgam 98.3% Composite 98.2% Metal-Cerami	CNN and KNN models achieved high accuracy in dental restoration classification, with the hybrid CNN-KNN model with potential for automated diagnostics
Suryawanshi and Behera, 2023, 28 India	Predict flexural strength (FS) and Vickers hardness (VH) of composites in chewing tobacco using ML	XGBoost, AdaBoost, random forest, KNN Approach: Supervised learning	Dental composites: Z250 (Microfill), 3 Nanofills: Z350 Translucent Z350 Dentin Tetric N-Ceram (12 numbers of data)	Flexural tests Vickers micro-hardness tester	Single train-test split (40% test data)	FS prediction: AdaBoost 99% XGBoost 99% RF 97% KNN 71.6% VH prediction: XGBoost 99% AdaBoost 98% RF 96.5% KNN 82.6%	ML effectively predicts composite properties, as demonstrated by AdaBoost and XGBoost's accurate predictions, showing potential for applications in dentistry
Suryawanshi and Behera, 2023, 29 India	Prediction of abrasive wear behavior of composites using ANN	ANN models Approach: Bayesian regularization (BR) Levenberg-Marquardt (LM) Scaled conjugate gradient (SCG)	Dental composites (72) Tetric N-Ceram (nanofill), Z350 Dentin Shade (nanofill) Z350 Translucent Shade (nano-fill) Z250 (micro-fill)	Wear test (pin-on-disc tribometer) Scanning electron microscopy (SEM) pH meter	Single train-test split (15% of data)	BR: 99.7% LM: 99.3% SCG: 98.1%	ANN model predicts wear where complex relationships elude mathematical equations. ANNs with BR accurately predict dental composite wear, providing a cost-effective alternative to experiments
Suryawanshi and Behera, 2024, India	ML models analysis of composite wear	Multi-layer perceptron (MLP) KNN XGBoost Approach: supervised learning	Dental composites (72) Tetric N-Ceram (nanofill), Z350 Dentin Shade (nanofill) Z350 Translucent Shade (nano-fill) Z250 (micro-fill)	Wear test (pin-on-disc tribometer) pH meter	Single train-test split (20% of data)	XGBoost: 99.9% MLP: 98.9% KNN: 86.2%	ML, especially XGBoost, effectively predicts dental composite wear, offering a promising tool for dental materials development
Takahashi et al, 2021, Japan	Developing a DL method to detect dental prosthesis and restorations from oral images based on color and material	DL model: YOLOv3 algorithm	Images of dental arches (1,904)	Object detection algorithm: Software: Python 3.5.2 Libraries: Keras library 2.2.4 Backend: TensorFlow 1.12.0	Single train-test split (20% of data)	Overall: 80% Dental prostheses 80% Dental composite 60%	DL, especially YOLOv3, effectively identified dental prostheses and restorations, showing accuracy for dental prostheses but not tooth-colored restorations due to their similarity to natural teeth. Multi-modal imaging may improve detection
Tejada-Casado et al, 2022, Spain, Romania	Evaluate PCA-based method accuracy for predicting color and reconstructing spectral reflectance of dental composites	Principal components analysis (PCA)	Disk-shaped dental resin composite (DRC) 25 enamel-dentine combinations/DRC Dentine composites: VITAPAN Excell VITAPAN Dentine VITA Physiodens Enamel composites: VITA Enamel (En)	PCA was evaluated using RMSE, GFC for spectral curves, and CIEDE2000 color difference (ΔE00) from measured spectral data	Single train-test split (5-sample training set: 20 samples; 9-sample training set: 16 samples)	Overall acceptability: 5-sample training set 96% 9-sample training set 99% Overall Perceptibility 5-sample training set 32.5% 9-samples training set 83.9%	PCA-based algorithms reliably predict layered dental resin composite colors. The training set selection determines prediction accuracy These advances show promise for materials innovation and dental clinical applications
Varshney et al, 2024, India	Color variation of various aesthetic restorative materials used in pediatric dentistry	ML decision trees: Chi-square automatic interaction detector (CHAID) Classification and regression tree (CART)	Restorative materials (200) Glass Ionomer Cement (GIC) Resin-modified glass ionomer cement (RMGIC) Microhybrid Composite resin Nanohybrid composite resin	Digital spectrophotometer (color measurement)	Single train-test split (30% of data)	Mean absolute error (MAE) for training: CHAID model 0.332 CART model 0.379	The CHAID model optimally predicted color change in restorative materials Restorative material and timing affect staining with minimal food impact. Beverages can affect dental restorations
Wang et al, 2023, United States	ML prediction of microtensile bond strength (µTBS) in dental adhesives and key chemical features	Models (9): Logistic Regression (LR) KNN SVM Decision Tree RF Extra Trees Gradient Boosting (GB) XGB MLP Approach: supervised machine learning	Commercial dental adhesives (180 µTBS values from 81 dental adhesives)	ML algorithms: scikit-learn package in Python	10-fold cross-validation (10% of data) Nested cross-validation	9-Feature data set: 0.72–0.80 4-key feature data set (MDP, pH, organic solvent, HEMA) 0.74–0.81	The study achieved 81% AI accuracy in categorizing dental adhesives using four key chemical features, demonstrating the potential application of machine learning

Abbreviations: ANN, artificial neural network; AdaBoost, adaptive boosting; CNN, convolutional neural network; DL, deep learning; FlexStr, flexural strength; GBDT, gradient boosting decision tree; GFC, goodness of fit; Hema, 2-hydroxyethyl methacrylate; KNN, K-nearest neighbors; LightGBM, light gradient boosting machine; LCUs, light curing units; LMT, logistic model trees; MDP, 10-methacryloyloxydecyl dihydrogen phosphate; ML, machine learning; MLP, multilayer perceptron; RF, random forest; RMSE, root mean square error; ShrinkStre, shrinkage stress; SVM, support vector machine; ShrinkV, volumetric shrinkage; XGBoost, extreme gradient boosting; YOLOv3, You Only Look Once version 3.

Note: Random forest, logistic regression, Gaussian Naïve Bayes, extreme learning machine (ELM), and extreme gradient boosting (XGBoost). Regression models: voting regressor, decision tree regression, voting regressor, and decision tree regression.

a ML classification algorithms: support vector machine (SVM), decision tree, K-nearest neighbors (KNN) classifier, and light gradient boosting machine (LGBM).

b Initially used: linear regression, polynomial regression, random forest, and support vector regression (SVR).

### Reporting and Transparency

Across the included AI/composite studies, TRIPOD–AI and STARD–AI were not used, code and fully open datasets were rarely shared, formal calibration analyses were uncommon, and no study reported a decision–curve or other quantitative clinical utility analysis, underscoring a substantial methodological gap in this field ( Supplementary Table S2 , available in the online version only).

### Quality and Risk of Bias Assessment

The risk of bias assessments is summarized in Supplementary Material (available in the online version only). In total, half of the studies ( *n* = 7) had a moderate risk of bias, 26 to 32, with six studies showing a low risk, 8.29 to 33, and only one study exhibiting a high risk of bias. 26 Seven studies were based on in vitro data and were evaluated using the QUIN risk-of-bias tool. 27 28 29 30 31 32 33 Despite three of these in vitro studies demonstrating strong internal validity through randomization and blinding, 30 31 32 the inherent limitations of in vitro research and the impact of operator skill suggest that the generalizability of the findings to routine clinical practice might be limited. Four studies focused on assessing the diagnostic accuracy of an AI model, with two showing moderate, 34 35 one low, 36 and one high risk of bias due to bias in patient selection, test conduct, and interpretation, as well as reference-standard subjectivity. 26 Three studies involved the development or validation of a prediction model, with two showing a low risk of bias 8 37 and one raising a risk of concern regarding applicability. 38

### Validation Approach and Data Size

A structured summary of dataset size, class balance, validation strategy (e.g., single hold-out split, k-fold cross-validation), and whether any external or multicenter validation was performed is provided for each included study in Supplementary Table S1 (available in the online version only). The dataset size showed considerable variation, which was attributed to the diverse inputs and outcomes across different study designs. The total sample sizes ranged from 1,223 to 10,220 across 8 datasets. In the included studies, most validation methods used a single-train-test split ( *n* = 10). At the same time, two studies integrated this approach with cross-validation, 26 34 and one study primarily used cross-validation. 37 In addition, two studies did not specify any validation method. 30 32 None of the studies provided formal calibration analyses or decision curve/clinical utility assessments, and only a minority explicitly referenced AI-specific reporting guidelines.

### Artificial Intelligence Approaches and Algorithms

**Fig. 2:**
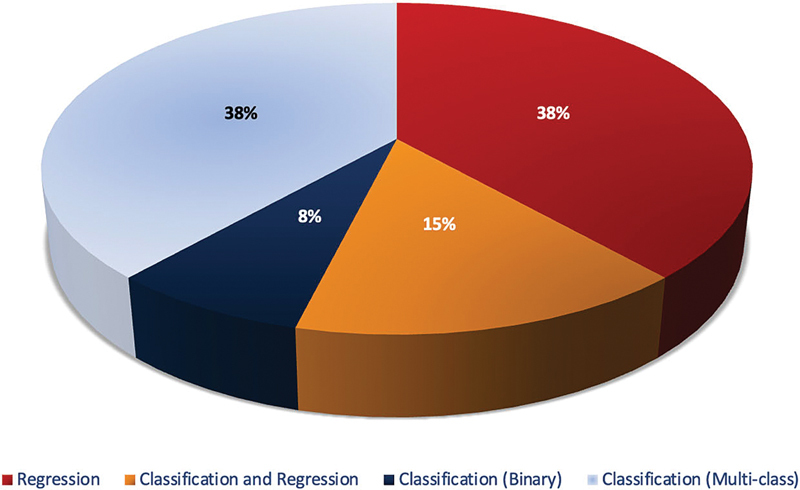
Supervised learning AI approach.

In all but one study, the AI algorithms used supervised learning, a Machine Learning strategy focused on classification, regression, or both ( Fig. 2 ). These algorithms were primarily composed of neural networks ( *n* = 10/13), with CNN (Convolutional Neural Networks) being the most common ( *n* = 5/13), 8 26 34 35 36 followed by ANN (artificial neural networks; *n* = 2/13) 29 32 and multilayer perception ( *n* = 2/13), 27 37 while one study utilized ELM (extreme learning machine). 38 Furthermore, five studies adopted feature extraction methods, 8 27 36 37 38 one implemented an object detection algorithm, 35 and another study combined both techniques. 26 Only one study applied an unsupervised learning approach, specifically utilizing PCA (principal component analysis) to reconstruct reflectance and estimate color. 33

### Performance Metrics Applied to Assess Accuracy

The accuracy of AI models applied in the included studies was predominantly > 80% for prediction modeling and composite characteristics evaluation. Although several studies reported very high apparent performance (often > 95–99% accuracy or area under the curve [AUC]), these values were typically obtained on relatively small, single-center datasets, frequently using a single train–test split and without independent external validation ( Supplementary Table S1 , available in the online version only). Specifically, very high performance was reported for several tasks: an ANN achieved 99.7% accuracy for predicting abrasive wear of composites in vitro, a combination of AdaBoost and XGBoost achieved up to 99% accuracy for predicting flexural strength and Vickers hardness, and MLP and XGBoost models reached 98.9 to 99.9% accuracy for predicting composite wear under simulated oral conditions. To evaluate the performance and accuracy of AI models in classifying dental restorations or predicting the properties of dental composites, a range of metrics was employed. These accuracy metrics measure various facets of model performance, with particular emphasis on the correlation between predicted outcomes and experimental data. In the context of classification tasks, the accuracy metrics used for this approach included accuracy ( *n* = 4), 26 34 37 38 precision ( *n* = 4), 8 26 34 38 recall/sensitivity ( *n* = 5), 8 26 34 36 38 specificity ( *n* = 2), 34 36 F1-score ( *n* = 3), 8 26 38 confusion matrices ( *n* = 2), 8 34 receiver operating characteristic curves, 8 AUC ( *n* = 4), 34 36 37 38 average precision, 35 mean average precision, 35 mean intersection over union, 35 overall diagnostic accuracy, 36 and MAE (mean absolute error). 31

The metrics for the regression approach in the included studies were alpha standard error, 32 MAE, 27 28 RMSE (root mean square error), 27 28 33 *R* ^2^ (coefficient of regression), 27 28 29 average absolute deviation percentage, 27 28 pseudo R-square, 30 sum square error, 29 mean relative error, 29 and MSE (mean square error). 29 The only study that applied unsupervised learning through PCA relied on a combination of spectral (RMSE, GFC [goodness of fit coefficient]) and color (ΔE _00_ : CIEDE2000 Formula) difference metrics for accuracy evaluation. The GFC was used to measure spectral curve shape similarity, and ΔE _00_ assesses color differences between actual and projected CIE Lab values. 33 These reported “highest accuracies” are task-specific for each model (detailed in Supplementary Table S1 , available in the online version only) and should not be interpreted as evidence that one algorithm is universally superior across all dental composite-related applications.

### Application of AI for Dental Composite Restoration

**Fig. 3:**
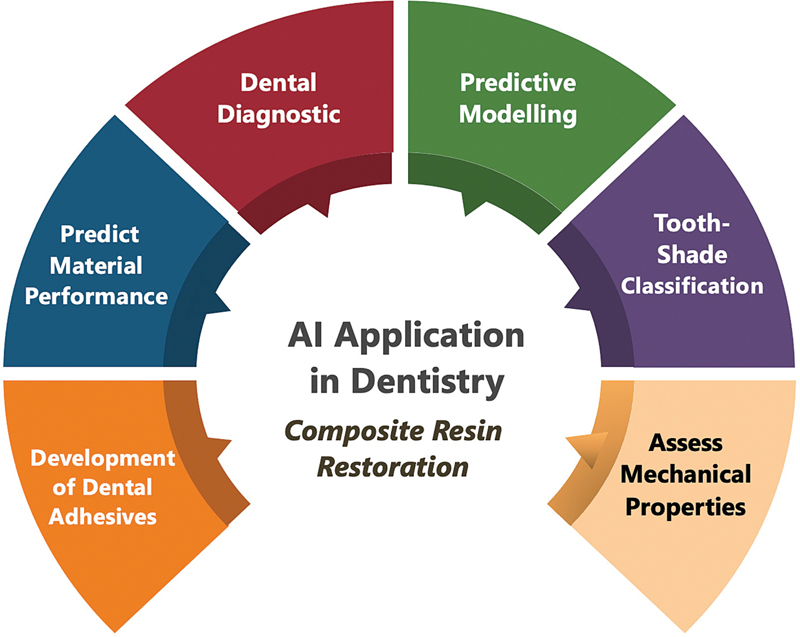
AI application in restorative dentistry.

The reviewed studies demonstrated broad applications of AI in dental composite evaluation, ranging from quantifying color change to predicting mechanical properties of dental composite restorations and differentiating tooth-colored composites from other dental restorations with high precision and reliability ( Fig. 3 ).

In these evaluations, the predominant AI application in the assessment of dental composites was identified as predictive modeling ( *n* = 9). This approach was employed to quantify color changes, 31 to estimate the color of layered dental composites, 33 and to automate an accurate shade-matching process. 26 AI algorithms were also used to predict performance outcomes of dental composites based on their attributes and optimize composite design by identifying the most effective attribute combinations. 38 AI algorithms also accurately predicted mechanical properties such as wear resistance, 27 flexural strength, and Vickers hardness, 28 as well as the abrasive wear behavior of dental composites. 29 Additionally, AI was applied to analyze and predict the depth of cure in composite restorations 32 and to predict the microtensile bond strength (µTBS) of dental adhesives based on their chemical characteristics, evaluating the potential for failure mechanisms like debonding. 37

Other applications of AI reported in reviewed studies included automated detection and classification of existing composite restorations, differentiation of composite from other restoration types using intraoral images 8 35 36 and from radiographic images 34 with high diagnostic accuracy. All diagnostic imaging studies relied on internal validation only, using single-center datasets with train–test splits and/or k-fold cross-validation. None performed external, multi-center validation or formally assessed cross-device generalization across different cameras or radiographic units, which restricts the applicability of their reported diagnostic performance. Furthermore, studies reported analysis of complex datasets for composite restoration evaluation, facilitating the identification of key variables influencing outcomes such as microleakage, 30 and depth of cure of composite restorations. 32

## Discussion

This is the first review to summarize the evidence for the application of AI in the evaluation of dental composite restorations. The studies under review utilized a range of AI methodologies, including CNNs, ANN, ELM, MLP, SVMs, XGBoost, AdaBoost, Random Forest, PCA, and Decision Trees. Most models achieved high accuracy, particularly ANN, XGBoost, and MLP for predicting dental composite mechanical properties; CNN-KNN hybrid for identifying dental restorations; and a PCA-based approach for predicting color differences, highlighting the adaptability of various AI techniques in dental composite contexts. Nevertheless, the exceptionally high accuracy and AUC values reported in some studies should be interpreted with caution. These findings are often derived from limited sample sizes, single-center data, single train–test partitions, and a lack of external validation, all of which increase the risk of overestimating performance and over-fitting. Taken together, these findings should be viewed primarily as proof–of–concept and technical feasibility demonstrations that AI can learn meaningful patterns in composite-related data, rather than as evidence that any specific model is ready for routine clinical application. Currently, therefore, the available models should be considered experimental instruments rather than systems prepared for clinical use, as none have yet been subjected to prospective, real-world evaluation or formal regulatory assessment.

The AI algorithms reported in the included studies demonstrated the potential to enhance diagnostic accuracy and treatment, 8 guiding clinical decision-making regarding light-curing techniques and material choices to ensure adequate polymerization and the longevity of restorations. 32 The effectiveness of restorative dental materials and the development of novel biocompatible alternatives require an in-depth knowledge of their mechanical characteristics. AI approaches, particularly Machine Learning algorithms, offer substantial benefits for assessing dental composites by enabling accurate predictions of mechanical properties from limited datasets. This leads to more streamlined analytical processes, expedited development of novel materials, and enhanced insight into material performance across dental applications.

Three studies by the same research group demonstrated the utility of an AI approach for accurately predicting the mechanical properties of dental composite materials. 27 28 29 In a controlled experimental study, the potential of a feed-forward back-propagation ANN model was explored for predicting abrasive wear of dental composites. 29 The findings indicated that this model serves as a robust and effective predictive tool for assessing material wear, significantly reducing errors linked to assumptions and demonstrating strong alignment with empirical data. As such, it offers a valuable alternative to more resource-intensive conventional methods. Notably, the combination of normal load and immersion duration in a tobacco solution had the most pronounced impact on wear metrics. 29

In a follow-up investigation by the same research team, the in vitro study successfully demonstrated the application of Machine learning algorithms through AdaBoost and XGBoost models to predict flexure strength and Vickers hardness of dental composite materials under simulated oral conditions, highlighting the potential of AI to optimize material evaluation and innovation in dentistry. 28 In another study that explored the application of Machine learning in predicting the wear characteristics of dental composites, the three distinct models of MLP, KNN, and XGBoost were utilized. 27 The findings revealed that both the MLP and XGBoost models exhibited superior accuracy and performance in wear prediction compared to the KNN model. Furthermore, the study identified immersion time in tobacco solution as the most influential input parameter on wear outcomes. This indicates that the exposure duration to a simulated oral environment is a crucial factor influencing the degradation of composite materials. 27 Both of these studies highlighted that the KNN model is particularly vulnerable to the presence of missing values and outliers, which can significantly impact its accuracy, and its efficiency is compromised by the computational burden involved in calculating distances between new and existing data points. 27 28

There is an increasing need for an automated system to evaluate intraoral conditions efficiently. Traditional methods of intraoral assessment often hinge on the skill and expertise of the dentist. AI-driven approaches offer more uniform and objective evaluations, providing critical data that can enhance the accuracy and efficiency of dental treatment planning. Machine learning and deep learning techniques, including the hybrid CNN-KNN model, CNN, and the object detection application YOLOv3 (You Only Look Once version 3), demonstrate significant capability in accurately predicting common dental restorations. These models are proficient at distinguishing composite restorations from other types with a high to moderate level of accuracy when analyzing intraoral images 8 35 36 or dental radiographic images 30. Several studies have demonstrated the potential utility of AI, particularly Deep Learning, in other areas of dentistry. such as application of Deep Learning based CNN for the detection and diagnosis of dental caries, 39 diagnosis of oral cancer, achieving accuracies comparable to human experts, 40 evaluating periodontal 41 and endodontic conditions. 42

Machine Learning has also been applied to predict color changes, 31 color estimation of layered dental composites, 33 and automate the accurate shade matching process. 26 A controlled study applied machine Learning decision tree algorithms to predict color changes in esthetic restorative materials in pediatric dentistry. 31 The findings indicate that restorative material selection and beverage consumption determine the esthetic stability of dental restorations in children. This study emphasizes choosing appropriate materials and considering patients' dietary habits, as these factors ensure long-term esthetic success. 31 In a separate controlled experimental study, the precision of a PCA-based algorithm was assessed to evaluate its ability to predict color properties of layered dental composites. 33 This research showed that PCA-based algorithms can accurately reconstruct the spectral reflectance of layered dental resin-based composites and predict their color with high precision, which was consistent across various material shades and thicknesses. 33 Manual dental shade matching lacks consistency and patient satisfaction. As an alternative to costly scanners, a mobile application using a CNN via MediaPipe detects facial landmarks and isolates teeth using an SVM to classify dental shades, achieving high accuracy. 26 The study shows a shift toward objective shade matching, benefiting clinicians and patients by integrating advanced technology.

Collectively, these findings indicate a promising trajectory for AI-driven color prediction in dentistry, facilitating more precise and aesthetically refined dental restorations through enhancing material design and manufacturing techniques.

### Challenges and Benefits of AI Algorithms

Table 2 provides a detailed overview of the primary AI model type, its essential function, limitations, and the potential applications associated with each AI algorithm reviewed in the included studies. Common challenges included limited availability of annotated datasets and inconsistent imaging modalities, both of which are essential for practical training and validation of AI models. 43 44 Small datasets often contain missing data and exhibit considerable complexity, which can lead to overfitting and limit the ability to generalize and make reliable predictions on new data. Future studies should aim to expand these datasets to improve predictive accuracy, as larger datasets would allow for more precise classification. Another challenge is distinguishing composite restorations from other restorations, which relies on color differences. While effective for metallic restorations, accurately detecting differences between ceramic, composite resin, and natural teeth is challenging.

**Table 2:** Benefits and limitations of AI models used in eligible studies

Study, year	Main type of AI model and core function	Benefits of an AI model	Limitations of the AI model
Almoro et al, 2024	ML algorithms: SVMs to classify final dental shades, DL-based CNN through the MediaPipe framework for Facial Landmark Detection	• Objective method for dental shade matching that reduces errors from observer fatigue and lighting • Improved accuracy and reliability • Enhanced patient esthetic outcomes • Accessible alternative to expensive intraoral scanners and other shade-matching devices • Convenient alternative to time-consuming visual assessment	• Limited training dataset size for certain shades • Limitations of F-Score for complex classification, as shade matching does not rely on binary data • The disparity between the system's internal accuracy (68.5%) and user testing (90%) reveals challenges in measuring AI against subjective assessment
Dilian and Kadhim, 2022	ML algorithm: Random forest model (RF) analytical tool to process data, identify key factors, and predict marginal microleakage in dental restorations	• Model identified the type of composite as a key factor influencing marginal microleakage • Predictive capability: compared predicted vs. actual microleakage values	• Sample Size Constraint: Limited sample size (84 observations and 500 trees) to generalize and make reliable predictions for new data • Larger datasets are needed in future research to improve its predictive accuracy
Engels et al, 2022	DL-based CNN to detect and categorize dental restorations from intraoral photographs	• High Diagnostic Accuracy > 90% • Automated Detection: reduces clinician workload • Efficiency: cost-effective procedure • Transfer Learning: Using a pretrained CNN on ImageNet accelerates training by leveraging existing learning results • Image Augmentation: Training data robustness improves model generalization	• Need for accuracy and practicality improvements • Direct composite fillings showed 92.9% diagnostic accuracy, with misclassifications between unrestored surfaces and composites due to color similarities • Requires images of less common restoration types for optimal performance • High accuracy needs time-intensive pixel-wise image annotation • Images with caries, developmental disorders, or sealants were excluded, indicating these could impact model performance
Karatas et al, 2021	DL-based CNN to detect, differentiate, and classify dental restorations from dental radiographs	• Promising technique for the detection and differentiation of restorations • High accuracy and specificity, even with small datasets • Overall classification success rate for different restorations > 90% • Reliable and accurate results • Automates classification of dental images containing different restorations • Effective edge detection • Minimize the effect of observer variability, light intensity, contrast, and brightness in identifying restorations on radiographs	• Sensitivity to restoration margins affecting reliability • Difficulty with similar opacity levels in restorations and classifying multiple restoration types in single radiographs (Larger datasets would enable more precise classification) • Higher resolution images would yield better distinctions • Common restorative materials like glass-ionomers and compomers were not included
Paniagua et al, 2025	ML algorithms (9): SVM for ShrinkStr classification KNN for FlexMod classification Decision tree for FlexStr and ShrinkV classification and regression analyses Logistic regression for ShrinkStr Random forest enhances generalization by constructing trees from different data subsets. XGBoost corrects errors from other models. Voting regressor combines predictions from multiple models to improve overall regression performance. Gaussian Naïve Bayes classification algorithm that assumes feature independence LGBM and ELM classification algorithms trained to predict composite performance outcomes	• Accelerated development: ML models predict material behavior from composite datasets, speeding development and improving clinical outcomes • AI models predict performance outcomes of experimental composites before fabrication, reducing testing time • Optimize composite design for durability • Enable nonlinear classification in computational materials • Can handle complex relationships without prior data distribution knowledge • Improve predictions by aggregating decision trees • Provide interpretability via splits and show feature impacts probabilistically	• Complex models can overfit when working with small datasets that contain missing data, leading Decision Trees to memorize the training set instead of finding patterns • Small samples result in poor generalization, and imputing missing values in these datasets can introduce bias • A single model cannot predict all performance outcomes due to differing variable-outcome relationships. Different models better predict specific POs, requiring multiple Machine Learning models • Error metrics vary as models with high *R* ^2^ values can still significantly differ from actual results
Rocha et al, 2022	ML algorithm: ANN model Analytical tool to identify the key variables influencing the depth of cure (DOC) in dental resin composites	• Ability to predict the DOC of resin-based composites • Effective in identifying the most crucial variables for predicting DOC • Capable of predicting DOC with high accuracy	• Predictions depend on input data. ANN can forecast DOC using average DOC and radiant exposure, showing these inputs' importance • Validation needed for other materials and curing conditions • Analysis covered RBC type, radiant exposure, and irradiance, but excludes chemical compositions and environmental factors affecting DOC
Shubham and Banerjee, 2024	ML algorithms (a hybrid approach) CNN for image classification KNN works with CNN to enhance classification by using feature maps for precise predictions	• CNN-KNN model achieved high overall accuracy (98.2%) • The model's accuracy in distinguishing dental restorations shows promise for improving diagnostic applications • High precision and recall values show reliability in differentiating restorations, improving diagnostics • Machine Learning models automate classification tasks • Training on a balanced dataset ensures effective generalization	• Model Misclassification: The model showed 60 false positives and negatives for restoration classifications, highlighting areas that require improvement • While CNNs may face issues identifying similar classes, KNN integration can help address this • Model performance depends on dataset quality and balance. While balanced data was used, comprehensive datasets remain essential for classification
Suryawanshi and Behera, 2023 28	ML algorithms (4): XGBoost to predict flexural strength and Vickers hardness AdaBoost to improve the performance of binary classifiers Random Forest improves precision and reduces overfitting by averaging results from trees KNN to identify the closest data points to predict from a query point	• Effective analysis of the mechanical properties of dental composites • AdaBoost and XGBoost showed high predictive accuracy for flexural strength and Vickers hardness prediction • Mechanical properties predicted using minimal data are valuable for resource-intensive testing • Identified complex relationships that traditional methods might miss, improving material understanding • XGBoost handles high-dimensional data robustly using patterns to predict material performance	• KNN has a significant drawback as it performs poorly in higher dimensions with increased complexity, and overall has lower accuracy compared to other models • Computationally expensive due to distance calculations between points • KNN requires handling of missing values and outliers before use • In general, a small dataset limits model generalizability and risks overfitting
Suryawanshi and Behera, 2023 29	Feed-forward back propagation ANN To predict dental material wear in microns analyzing wear characteristics based on parameters like material type, immersion days, load, speed, and track width	• Ability to predict dental composite wear • Cost-effective alternative to experiments with faster training • High accuracy and reduces error • Experimental data reduces modeling errors • ANNs excel when mathematical equations cannot describe nonlinear relationships between variables • Bayesian regularization training enhances generalization for limited/noisy datasets	• Training requirements: Neural networks require offline training for predictions. Training updates ANN weights to minimize errors • Data dependency: ANN models need adequate input datasets for effectiveness. Pretraining enables accurate dataset interpretation • Computational resources: Bayesian regularization is time-consuming
Suryawanshi and Behera, 2024	ML algorithms (3): MLP to decipher input-output links by evaluating weighted combinations across layers with activation functions, enabling MLP to handle noisy data. XGBoost provides robust predictive capability through ensemble learning. KNN offers distance-based classification or regression	• Effectively predict dental composite wear properties • MLP learns complex input-output relationships through nonlinear activation and hidden layers, while being resilient to noisy data and input parameter variations	• KNN underperformed vs. MLP and XGBoost due to distance-based computation costs and sensitivity to outliers • The pin-on-disc tribometer did not fully replicate oral conditions, excluding temperature effects and using constant speeds • A larger sample would improve predictions
Tejada-Casado et al, 2022	Principal component analysis (PCA) to reconstruct spectral reflectance data and estimate the color of layered dental resin-based composites (DRC) with varying thicknesses	• Simplicity and versatility as a dimensionality-reduction method for spectral reconstruction • Effectively reconstruct spectral reflectance and estimate color within acceptable thresholds • Accurately predicted spectral reflectance of layered dental resin-based composites with varying shades and thicknesses • Method accuracy improved with more training samples	• Performance depends on the type of composite used • Diamond configuration showed less shade dependence than X-shaped, which declined with darker shades • Despite better performance, the diamond configuration lacks input for extreme thickness, potentially limiting prediction • The study was limited to composite materials and specific shades, requiring future research with more materials • Using only two layers is a limitation, though sufficient for direct restorations clinically
Takahashi et al, 2021	DL object detection model: You Only Look Once version 3 (YOLOv3) to identify dental prostheses and restorations from oral images to provide quicker and consistent assessment of intraoral conditions	• High accuracy in detecting metallic dental prostheses, with average precision for composite resin restoration • The system demonstrated an 80% success rate in identifying splinted vs. unsplinted prostheses, highlighting its capability to recognize structural variations • Streamlined intraoral processing reduces time, and the dentist's expertise is needed • Good performance for object detection	• Only 60% of tooth-colored prostheses were correctly identified • Recognition relies on color differences, which works for metallic items but makes accurate detection difficult between ceramic, composite resin, and natural teeth • Tooth-colored prostheses were often misdiagnosed as sound teeth due to their similar appearance • The study's 1,900 images were insufficient to recognize all prosthesis types used in clinical practice • The algorithm cannot identify sound teeth effectively • System performance is limited with oral photographs alone. Combining with X-ray and digital scanning may improve accuracy
Varshney et al, 2024	ML algorithms: Decision tree CHAID and CART models to create a model for predicting factors causing color changes in dental restorations, and determine how variables such as material type, food, or time can explain color change	• Predictive modeling: develops models to determine variables' effects on outcomes, predicting color changes • Factors influencing color change with restorative material types emerged as key predictors, followed by time interval, food items, and polished status • Models identify factors affecting dental restoration color stability offer clinical insights • The CHAID model showed higher accuracy with lower mean absolute error than the CART, making it preferable for color change prediction	• While decision trees are considered interpretable, their complexity with multiple splits/nodes can be challenging to understand without detailed analysis • As an in vitro study, the predictions are based on controlled laboratory conditions, not accounting for the oral environment, masticatory forces, or pH levels • Clinical applicability is limited without in vivo validation • Accuracy relies on input data quality and representativeness, with biases potentially affecting predictions
Wang et al, 2023	ML algorithms (9): Logistic regression, KNN, SVM, decision trees, Tree-based ensembles (random forest, extra trees, gradient boosting, extreme gradient boosting) Multilayer perceptron (MLP) Function: prediction of microtensile bond strength and identification of important contributing factors	• Accelerates dental adhesive development by analyzing large datasets • Simultaneously identifies multiple contributing factors: MDP, pH, organic solvent (OS), and HEMA identified as key contributors to microtensile bond strength • Prediction of dental adhesive microtensile bond strength based on chemical features • Identifies optimal input patterns for dental adhesives • Computational predictions with experimental validation reduce time and costs	• Dental material datasets are small and complex, often leading to overfitting, where models have difficulty with new data • Feature selection can mitigate overfitting by reducing training data dimensionality • Tree-based ensemble methods were used with nested cross-validation to estimate model generalization • Ensemble models require more computational power than Logistic Regression and SVM, balancing complexity with performance

Abbreviations: ANN, artificial neural network; AdaBoost, adaptive boosting; CART, classification and regression tree; CHAID, chi-square automatic interaction detector; CNN, convolutional neural network; DL, deep learning; ELM, extreme learning machine; FlexStr, flexural strength; GBDT, gradient boosting decision tree; GFC, goodness of fit; Hema, 2-hydroxyethyl methacrylate; KNN, K-nearest neighbors; LGBM, light gradient boosting machine; LCUs, light curing units; LMT, logistic model trees; MDP, 10-methacryloyloxydecyl dihydrogen phosphate; ML, machine learning; MLP, multilayer perceptron; RCB, resin composite blocks; RF, random forest; RMSE, root mean square error; ShrinkStre, shrinkage stress; SVM, support vector machine; ShrinkV, volumetric shrinkage; XGBoost, extreme gradient boosting; YOLOv3, You Only Look Once version 3.

AI technology offers several important benefits, including increased accuracy and reliability, enhanced aesthetic outcomes for patients, and serving as a practical alternative to the lengthy visual assessment process, which reduces the workload of clinicians. Furthermore, AI can minimize the impact of observer variability and optimize composite design for greater durability. Additionally, integrating computational predictions with experimental validation streamlines processes and provides a cost-effective alternative to expensive intraoral scanners and other shade-matching devices, with potential to enhance diagnostic applications. Models have further identified factors that affect the color stability of dental restoration as well as key factors influencing marginal microleakage.

### Implications for Research and Practice

The current body of evidence, therefore, primarily represents proof-of-concept research demonstrating the potential of AI when applied in controlled environments, rather than models ready for adoption into routine restorative practices. Future studies should address regulatory requirements for AI-based medical devices and evaluate how these tools can be safely integrated into real-world clinical workflows and information systems. The integration of AI into research offers significant opportunities to enhance future studies and practical applications in dentistry. Machine Learning is gaining traction as a method for constructing predictive models and has been examined in various studies reviewed in this analysis. Its superior ability to manage complex, high-dimensional data holds the potential for integrating genetic and clinical predictors. Future research should focus on comparing machine Learning techniques with traditional methods and on testing models prospectively in clinical settings.

The AI models demonstrate strong capability to classify dental restorations and predict composite properties, suggesting a future transition toward more objective, efficient, and personalized diagnostic planning in dentistry, 8 provided that robust external validation and implementation studies confirm their performance and safety in real-world use. The approach is a significant contribution to the expanding field of Machine Learning in dental diagnostics, as it helps identify intricate patterns in dental images, particularly those related to composite restorations. Furthermore, such development in AI tools could save dentists time and diminish inter-examiner variability, with the potential to become a standard part of routine clinical assessment. Integrating data from intraoral camera systems into AI models would enable immediate evaluation of dental restorations, thereby optimizing the diagnostic workflow. 36 Machine Learning models, such as AdaBoost, XGBoost, and MLP, provide a robust approach for predicting composite material mechanical characteristics. This methodology can expedite the development of dental materials by delivering precise mechanical prediction without relying on physical tests. Furthermore, the AI approach identifies key factors affecting dental composite properties that can be used in future research to develop materials with improved resistance to environmental exposure, and more focused study designs optimizing experimental setups. The success of ML in predicting composite properties highlights its potential for broader applications in dental material science and clinical dentistry, such as predicting wear behavior, biocompatibility, and the long-term clinical success of restorations. 27 28 29 Insights from large dental imaging benchmarks highlight the importance of high-quality, multi-center annotations for restorations, yet current datasets rarely distinguish composite from other tooth–colored materials. 17 19 20 Future composite-focused AI work should align with these benchmarks by integrating material-level labels and harmonized imaging protocols. 19 20

The predictive modeling capabilities of AI further enable clinicians to forecast the lifespan and potential failure risks of composite materials. This capability supports evidence-based treatment planning and allows for the personalized selection of materials, hence optimizing the durability of restorations and enhancing patient satisfaction. 45

## Strengths and Limitation

The key strengths of this review included comprehensive searches of multiple databases, and the majority of studies were of moderate to low risk of bias. In addition, all studies reported robust model evaluation metrics, with more than half using multiple Machine Learning models to identify the most effective AI approach for evaluating composite materials. This study acknowledges several limitations. Primarily, as a scoping review, it offers a general overview of the current use of AI in dental composite restoration and identifies areas where more research is needed. However, unlike a systematic review, it does not confirm the reliability of the existing knowledge. The limited number of studies included in the present scoping review may be attributed to the relatively recent emergence of AI applications within the field of dentistry over the past few decades. Furthermore, the majority of the articles reviewed were in vitro studies that often did not include randomization or blinding. This lack of methodological rigor limits the generalizability of the results and could be addressed through external validation. Consequently, despite encouraging internal performance, the included models should be considered at an early developmental stage, and none can yet be regarded as clinically validated decision-making tools for everyday practice. This structured assessment further highlights key gaps in current AI research on composite restorations: rare use of AI-specific reporting guidelines, limited sharing of code and reusable datasets, infrequent formal calibration, and a lack of quantitative clinical utility evaluations such as decision–curve analysis, all of which hamper reproducibility and translation into practice. Future AI studies in restorative dentistry should therefore follow AI-specific reporting guidelines, share code and data where feasible, and incorporate formal calibration and clinical utility analyses to enable robust validation and safe clinical application. Although the review protocol was not prospectively registered, detailed search strategies, screening forms, and data–extraction templates are available in Supplementary Material (available in the online version only) to enhance transparency. A further limitation is the complete absence of external, multi-center validation and cross-device generalization analyses in the included diagnostic AI studies. All performance estimates were therefore based on internal validation within single-center datasets, which likely overestimates clinical robustness. This pattern is consistent with optimism in internal performance estimates noted earlier and highlights the need for larger, externally validated datasets before clinical implementation is considered.

## Conclusion

This scoping review represents the first comprehensive examination of AI models for evaluating dental composite restorations. Several models are currently available, many of which demonstrate moderate to excellent predictive capabilities. However, most studies assessing these models were uniformly at risk of bias, primarily due to limited datasets and small sample sizes, resulting in poor generalizability. Consequently, the reported performance metrics of these models are likely overly optimistic and may not be replicable in clinical settings. Taken together, the available evidence indicates that existing AI models for composite restorations remain at a nascent, exploratory phase and should not yet be regarded as suitable or routine clinical decision-making. At present, their application is confined to experimental evaluation and hypothesis generation rather than offering direct support in patient care. While this review does not promote any specific model for clinical application, it compiles existing AI models, their performance during internal validation, and the sample sizes associated with them. This compilation highlights key methodological gaps, such as small, single-center datasets, a lack of external validation, and limited assessment of clinical utility, and is intended to guide future work in developing, validating, and, when robustly supported, ultimately translating AI models for composite restorations into practice.
