# Supplementary material for: Emerging Artificial Intelligence Technologies for Evaluation of Dental Composite Restorations: A Scoping Review
Source: Eur J Dent. 2026 Feb 12;20(3):704–20. doi: 10.1055/s-0046-1816536 (PMC13337272; doi:10.1055/s-0046-1816536)
Supplement: Supplementary file 1 — Supplementary Material [file 10-1055-s-0046-1816536-s25114663.pdf]

Preferred Reporting Items for Systematic Reviews and Meta-Analyses extension for Scoping Reviews (PRISMA-ScR) checklist

SECTION	ITEM	PRISMA-ScR CHECKLIST ITEM	REPORTED ON PAGE #
TITLE			
Title	1	Identify the report as a scoping review.	Title page
ABSTRACT			
Structured summary	2	Provide a structured summary that includes (as applicable): background, objectives, eligibility criteria, sources of evidence, charting methods, results, and conclusions that relate to the review questions and objectives.	1
INTRODUCTION			
Rationale	3	Describe the rationale for the review in the context of what is already known. Explain why the review questions/objectives lend themselves to a scoping review approach.	2
Objectives	4	Provide an explicit statement of the questions and objectives being addressed with reference to their key elements (e.g., population or participants, concepts, and context) or other relevant key elements used to conceptualize the review questions and/or objectives.	3
METHODS			
Protocol and registration	5	Indicate whether a review protocol exists; state if and where it can be accessed (e.g., a Web address); and if available, provide registration information, including the registration number.	3,4
Eligibility criteria	6	Specify characteristics of the sources of evidence used as eligibility criteria (e.g., years considered, language, and publication status), and provide a rationale.	4
Information sources[a]	7	Describe all information sources in the search (e.g., databases with dates of coverage and contact with authors to identify additional sources), as well as the date the most recent search was executed.	4
Search	8	Present the full electronic search strategy for at least 1 database, including any limits used, such that it could be repeated.	Supp File 2
Selection of sources of evidence[b]	9	State the process for selecting sources of evidence (i.e., screening and eligibility) included in the scoping review.	4,5
Data charting process[c]	10	Describe the methods of charting data from the included sources of evidence (e.g., calibrated forms or forms that have been tested by the team before their use, and whether data charting was done independently or in duplicate) and any processes for obtaining and confirming data from investigators.	5,6
Data items	11	List and define all variables for which data were sought and any assumptions and simplifications made.	NA
Critical appraisal of individual sources of evidence[d]	12	If done, provide a rationale for conducting a critical appraisal of included sources of evidence; describe the methods used and how this information was used in any data synthesis (if appropriate).	6
Synthesis of results	13	Describe the methods of handling and summarizing the data that were charted.	6
RESULTS			
Selection of sources of evidence	14	Give numbers of sources of evidence screened, assessed for eligibility, and included in the review, with reasons for exclusions at each stage, ideally using a flow diagram.	6
Characteristics of sources of evidence	15	For each source of evidence, present characteristics for which data were charted and provide the citations.	6
Critical appraisal of sources of evidence	16	If done, present data on critical appraisal of included sources of evidence (see item 12).	7
Results of individual sources of evidence	17	For each included source of evidence, present the relevant data that were charted that relate to the review questions and objectives.	6–11

 © 2026. The Author(s).

(*Continued*)

SECTION	ITEM	PRISMA-ScR CHECKLIST ITEM	REPORTED ON PAGE #
Synthesis of results	18	Summarize and/or present the charting results as they relate to the review questions and objectives.	6–11
DISCUSSION			
Summary of evidence	19	Summarize the main results (including an overview of concepts, themes, and types of evidence available), link to the review questions and objectives, and consider the relevance to key groups.	11
Limitations	20	Discuss the limitations of the scoping review process.	17–19
Conclusions	21	Provide a general interpretation of the results with respect to the review questions and objectives, as well as potential implications and/or next steps.	19
FUNDING			
Funding	22	Describe sources of funding for the included sources of evidence, as well as sources of funding for the scoping review. Describe the role of the funders of the scoping review.	20

Abbreviations: JBI, Joanna Briggs Institute; PRISMA-ScR, Preferred Reporting Items for Systematic reviews and Meta-Analyses extension for Scoping Reviews.
[a]Where *sources of evidence* (see second footnote) are compiled from, such as bibliographic databases, social media platforms, and websites.
[b]A more inclusive/heterogeneous term used to account for the different types of evidence or data sources (e.g., quantitative and/or qualitative research, expert opinion, and policy documents) that may be eligible in a scoping review as opposed to only studies. This is not to be confused with *information sources* (see first footnote).
[c]The frameworks by Arksey and O'Malley (6) and Levac and colleagues (7) and the JBI guidance (4, 5) refer to the process of data extraction in a scoping review as data charting.
[d]The process of systematically examining research evidence to assess its validity, results, and relevance before using it to inform a decision. This term is used for items 12 and 19 instead of "risk of bias" (which is more applicable to systematic reviews of interventions) to include and acknowledge the various sources of evidence that may be used in a scoping review (e.g., quantitative and/or qualitative research, expert opinion, and policy documents).
Source: Tricco AC, Lillie E, Zarin W, O'Brien KK, Colquhoun H, Levac D, et al. PRISMA Extension for scoping reviews (PRISMAScR): checklist and explanation. Ann Intern Med. 2018;169:467–473. doi: 10.7326/M18-0850.

Supplementary Table S1 Search strategy in PubMed (2020–2025)

No.	Key term searches	Results
#1	"artificial intelligence" OR "machine learning" OR "3D Printing" OR "3D technology" OR "Robotic dentistry" OR "fuzzy decision" OR "neural network*" OR "deep learning" OR "Natural Language Processing" OR "digital dentistry" OR "advanced technolog*" OR "additive manufacturing" OR "subtractive manufacturing" OR "Artificial Intelligence"[Mesh] OR "Machine Learning"[Mesh]	569,765
#2	"resin-based composite*" OR "dental resin composite*" OR "composite restoration*" OR "resin composite*" OR "restorative dental composite" OR "Composite Resins"[Mesh]	32,648
#3	#1 AND #2	1,145
#4	#3 AND Filter (last 5 years)	589

Supplementary Table S2 Search strategy in Embase (2020–2025)

No.	Key term searches	Result
1	("artificial intelligence" or "machine learning" or "3D Printing" or "3D technology" or "Robotic dentistry" or "fuzzy decision" or "neural network*" or "deep learning" or "Natural Language Processing" or "digital dentistry" or "advanced technolog*" or "additive manufacturing OR Computer-Aided Design").mp. [mp = title, abstract, heading word, drug trade name, original title, device manufacturer, drug manufacturer, device trade name, keyword heading word, floating subheading word, candidate term word]	524484

(*Continued*)

 © 2026. The Author(s).

Supplementary Table S2 (*Continued*)

No.	Key term searches	Result
2	("resin-based composite" or "dental resin composite*" or "resin-based composite" or "composite restoration*" or "resin composite*" or "restorative dental composite" or "Composite Resins").mp. [mp = title, abstract, heading word, drug trade name, original title, device manufacturer, drug manufacturer, device trade name, keyword heading word, floating subheading word, candidate term word]	13302
3	1 and 2	94
4	3 and 2020:2025.(sa_year).	83

Supplementary Table S3 Search strategy in Scopus (2020–2025)

No.	Key term searches	Result
5	(TITLE-ABS-KEY ("resin-based composite" OR "dental resin composite*" OR "resin-based composite" OR "composite restoration*" OR "resin composite*" OR "restorative dental composite" OR "Composite Resin*")) AND (TITLE-ABS-KEY ("artificial intelligence" OR "machine learning" OR "3D Printing" OR "3D technology" OR "Robotic dentistry" OR "fuzzy decision" OR "neural network*" OR "deep learning" OR "Natural Language Processing" OR "digital dentistry" OR "advanced technolog*" OR "additive manufacturing" OR "Computer-Aided Design")) AND PUBYEAR > 2019 AND PUBYEAR < 2026 AND (LIMIT-TO (DOCTYPE , "ar") OR LIMIT-TO (DOCTYPE , "re"))	1,018 results
4	(TITLE-ABS-KEY ("resin-based composite" OR "dental resin composite*" OR "resin-based composite" OR "composite restoration*" OR "resin composite*" OR "restorative dental composite" OR "Composite Resin*")) AND (TITLE-ABS-KEY ("artificial intelligence" OR "machine learning" OR "3D Printing" OR "3D technology" OR "Robotic dentistry" OR "fuzzy decision" OR "neural network*" OR "deep learning" OR "Natural Language Processing" OR "digital dentistry" OR "advanced technolog*" OR "additive manufacturing" OR "Computer-Aided Design")) AND (LIMIT-TO (DOCTYPE , "ar") OR LIMIT-TO (DOCTYPE , "re"))	1,634 results
3	(TITLE-ABS-KEY ("resin-based composite" OR "dental resin composite*" OR "resin-based composite" OR "composite restoration*" OR "resin composite*" OR "restorative dental composite" OR "Composite Resin*")) AND (TITLE-ABS-KEY ("artificial intelligence" OR "machine learning" OR "3D Printing" OR "3D technology" OR "Robotic dentistry" OR "fuzzy decision" OR "neural network*" OR "deep learning" OR "Natural Language Processing" OR "digital dentistry" OR "advanced technolog*" OR "additive manufacturing" OR "Computer-Aided Design"))	1,715 results
2	TITLE-ABS-KEY ("resin-based composite" OR "dental resin composite*" OR "resin-based composite" OR "composite restoration*" OR "resin composite*" OR "restorative dental composite" OR "Composite Resin*")	44,232 results
1	TITLE-ABS-KEY ("artificial intelligence" OR "machine learning" OR "3D Printing" OR "3D technology" OR "Robotic dentistry" OR "fuzzy decision" OR "neural network*" OR "deep learning" OR "Natural Language Processing" OR "digital dentistry" OR "advanced technolog*" OR "additive manufacturing" OR "Computer-Aided Design")	3,001,398 results

Supplementary Table S4 Search strategy in Web of Science (2020–2025)

No.	Search query	Results
1	“artificial intelligence” OR “machine learning” OR “3D Printing” OR “3D technology” OR “Robotic dentistry” OR “fuzzy decision” OR “neural network*” OR “deep learning” OR “Natural Language Processing” OR “digital dentistry” OR “advanced technolog*” OR “additive manufacturing“ OR "Computer-Aided Design" (All Fields)	2,241,606
2	“resin-based composite” OR “dental resin composite*” OR “resin-based composite” OR “composite restoration*” OR “resin composite*” OR “restorative dental composite” OR "Composite Resin*" (All Fields)	27,058
3	#1 AND #2	892
4	#1 AND #2	892

 © 2026. The Author(s).

Supplementary Table S4 (*Continued*)

No.	Search query	Results
5	#1 AND #2 and Book Chapters or Editorial Material or Correction (Exclude – Document Types)	889
6	#1 AND #2 and Book Chapters or Editorial Material or Correction (Exclude – Document Types) and 2020 or 2021 or 2022 or 2023 or 2024 or 2025 (Publication Years)	652
7	#1 AND #2 and Book Chapters or Editorial Material or Correction (Exclude – Document Types) and 2020 or 2021 or 2022 or 2023 or 2024 or 2025 (Publication Years) and Article or Review Article or Early Access or Proceeding Paper (Document Types)	652

Supplementary Table S5 Summary of all database searches before and after deduplication

No.	Databases	All	Other dup	After dups
1	PubMed	589		589
2	Embase	83	64	19
3	Scopus	1018	487	531
4	Web of Science	652	483	169
Total databases		2342	1034	1308

Structured Screening Form to Align with PRISMA–ScR and Eligibility Criteria

Title and Abstract Screening Form

Study ID:
Reviewer:
Date:

Topic Relevance
Does the study involve dentistry?

☐ Yes
☐ No → Exclude (Reason: non-dental)

Does the study involve *restorative* dentistry or dental materials?

☐ Yes
☐ No → Exclude (Reason: not restorative/materials)

Does the study mention composite resin/resin-based restorations or tooth–colored restorative materials?

☐ Yes
☐ Unclear
☐ No → Exclude (Reason: no composite–resin focus)

Artificial Intelligence/Machine Learning
Does the study apply artificial intelligence or machine learning methods (e.g., CNN, ANN, SVM, random forest, XGBoost, PCA)?

☐ Yes
☐ Unclear
☐ No → Exclude (Reason: no AI/ML)

Study Type and Data
Is the study empirical (in vitro, in vivo, experimental, or observational) rather than an opinion piece, editorial, letter, commentary, or review only?

☐ Yes
☐ No/opinion–only → Exclude (Reason: non-empirical)

 © 2026. The Author(s).

Is there any indication that numerical or measurable outcomes are reported (e.g., accuracy, strength, wear, colour change, bond strength)?

□ Yes
□ Unclear
□ No → Exclude (Reason: no measurable outcomes)

Provisional decision (title/abstract)

□ Include for full–text review
□ Exclude at the title/abstract screening

Reason for exclusion (tick all that apply):

□ Not dental
□ Not restorative/composite–related
□ No AI/ML
□ Non-empirical (opinion, letter, commentary)
□ No measurable outcomes
□ Other (specify): ______________________________

Full–text Screening Form

Study ID:
Reviewer:
Date:

Population/Material

A1. Does the study focus on **resin-based composite restorations or composite materials** relevant to permanent restorations?

□ Yes
□ Partly (mixed materials, but composite data separable)
□ No → Exclude (Reason: no relevant composite data)

A2. Are temporary materials, hybrid ceramics, ceramics, or dentures the **primary** focus?

□ Yes → Exclude (Reason: non-composite permanent materials)
□ No

A3. Are restorations labelled only as “fillings” or “restorations” without specifying composite, and can composite not be separated from other materials?

□ Yes → Exclude (Reason: composite not distinguishable)
□ No
□ Composite clearly separable (e.g., subgroup analysis)

AI/ML Intervention

B1. Does the study implement an AI or ML method (e.g., supervised or unsupervised learning, CNN, ANN, SVM, RF, XGBoost, PCA, etc.) as a core component of the analysis?

□ Yes
□ No → Exclude (Reason: no AI/ML)

B2. Is the AI/ML model applied to at least one of the following composite-related tasks?

□ Prediction of mechanical properties (wear, flexural strength, hardness, bond strength, etc.)
□ Shade matching or colour prediction/evaluation
□ Classification or detection of composite restorations in images/radiographs
□ Prediction of cure depth, microleakage, or other performance outcomes
□ Other composite–specific evaluation (specify): _______________________
□ None → Exclude (Reason: AI not used for composite evaluation)

 © 2026. The Author(s).

Outcomes and Measurability

C1. Are the main outcomes **numerical or measurable** (e.g., accuracy, AUC, RMSE, R^2, strength, wear depth, color difference, μTBS)?

☐ Yes
☐ No → Exclude (Reason: outcomes not measurable)

C2. Are the datasets and comparison benchmarks (expert opinion or reference standard) clearly described?

☐ Yes
☐ Partly (limited detail but usable)
☐ No → Consider exclusion or rate as high risk of bias

Study Design and Type

D1. Study setting (tick all that apply):

☐ In vitro
☐ In vivo (clinical)
☐ Ex vivo/extracted teeth
☐ Image/radiograph dataset only
☐ Other (specify): ________________

D2. Study type:

☐ Experimental
☐ Observational/diagnostic accuracy
☐ Prediction model development/validation
☐ Other: ________________

D3. Publication type:

☐ Full original article
☐ Conference abstract only → Exclude
☐ Thesis/dissertation → Exclude
☐ Other (specify): ________________

Language and Accessibility

E1. Is the full text accessible, and can methods and outcomes be reliably assessed (with translation support if needed)?

☐ Yes
☐ No → Exclude (Reason: full–text not assessable)

Eligibility Decision (Full Text)

1. ☐ Include in scoping review
2. ☐ Exclude

If excluded, tick **main reason(s)**:

☐ Not composite–resin or composite not distinguishable
☐ No AI/ML intervention
☐ No measurable outcomes
☐ Non-empirical/conference abstract/thesis only
☐ Full text not accessible/not assessable
☐ Other (specify): ________________________

Reviewer Comments/Notes:

Data extraction form summarizing reporting, transparency, and clinical utility across studies.

Study ID:
First author, year:

 © 2026. The Author(s).

Journal:
Reviewer/Date:

1. Bibliographic and general information
   1.1 Country/region of study:
   1.2 Study type (tick all that apply):
   - ☐ In vitro experimental
   - ☐ In vivo clinical
   - ☐ Ex vivo/extracted teeth
   - ☐ Image/radiograph dataset only
   - ☐ Prediction model development/validation
   - ☐ Diagnostic accuracy study
   - ☐ Other (specify): ___________________

   1.3 Main focus of study (free text, 1–2 lines):
   (e.g., "detect composite restorations on radiographs," "predict flexural strength and hardness," "automated shade matching")
2. Population/material and setting
   2.1 Sample type:
   - ☐ Teeth/restorations (clinical)
   - ☐ Extracted teeth
   - ☐ Composite specimens (blocks, discs, etc.)
   - ☐ Adhesives/materials only
   - ☐ Radiographic images (periapical/bitewing/panoramic)
   - ☐ Intraoral photographs
   - ☐ CBCT/other imaging
   - ☐ Other: ___________________

   2.2 Composite/material type(s) (name and category):
   (e.g., "nanohybrid composite, Tetric N–Ceram; Microhybrid composite …")

   2.3 Sample size:
   Total n: ______
   For imaging: number of images/teeth/restorations (specify unit): ______
   For specimens: number of specimens: ______

   2.4 Class balance (if applicable):
   (e.g., composite vs other restorations; categories and counts/percentages)
3. AI/ML methods
   3.1 AI/ML approach (tick all that apply):
   - ☐ Supervised learning – classification
   - ☐ Supervised learning – regression
   - ☐ Unsupervised learning (e.g., PCA)
   - ☐ Deep learning (CNN, etc.)
   - ☐ Hybrid methods (specify): _____________

   3.2 Specific algorithms/models used (list):
   (e.g., CNN, ANN, SVM, Random Forest, XGBoost, YOLOv3, PCA, etc.)

   3.3 AI task(s) addressed (tick all that apply):
   - ☐ Detection/classification of composite restorations
   - ☐ Differentiation of composite vs other materials
   - ☐ Shade matching/colour prediction
   - ☐ Prediction of mechanical properties (wear, flexural strength, hardness, etc.)
   - ☐ Prediction of cure depth
   - ☐ Prediction of microleakage or marginal integrity
   - ☐ Prediction of bond strength (e.g., μTBS)
   - ☐ Other (specify): ___________________

 © 2026. The Author(s).

4. Outcomes and performance metrics
   4.1 Primary outcomes (brief description):
   (e.g., "classification accuracy for composite vs other restorations", "RMSE for colour prediction", "R^2 for flexural strength")

   4.2 Performance metrics reported (tick all that apply):
   • Classification:
   o ☐ Accuracy
   o ☐ Precision
   o ☐ Recall/Sensitivity
   o ☐ Specificity
   o ☐ F1-score
   o ☐ AUC/ROC
   o ☐ AP/mAP/mIoU
   o ☐ Confusion matrix
   o ☐ Other: ___________

   • Regression:
   o ☐ MAE
   o ☐ RMSE
   o ☐ R^2/pseudo R^2
   o ☐ SSE/MSE/MRE/AAD%
   o ☐ Other: ___________

   • Colour/spectral:
   ☐ ΔE (e.g., ΔE_{00})
   ☐ RMSE (spectral)
   ☐ GFC
   ☐ Other: ___________
   4.3 Best–reported model performance (with context):
   (e.g., "XGBoost: accuracy 99% for predicting flexural strength ($n = 12$ specimens)")
5. Validation strategy and data sources
   5.1 Validation type (tick all that apply):
   ☐ Single train–test split (specify split, e.g., 80/20)
   ☐ k–fold cross–validation (k = _____)
   ☐ Nested cross-validation
   ☐ Bootstrapping
   ☐ External validation on an independent dataset
   ☐ Not clearly reported

   5.2 Data origin and centres:
   ☐ Single–centre
   ☐ Multi-centre (number of centres: ___)
   ☐ External multi-centre test set used: ☐ Yes / ☐ No

   5.3 Cross-device/cross-scanner analysis reported?
   ☐ Yes (describe devices and findings): ____________________
   ☐ No
6. Risk of bias and reporting
   6.1 Risk of bias tool applied in this review (for this study):
   ☐ QUIN
   ☐ QUADAS–2
   ☐ PROBAST
   ☐ Not applicable

   6.2 Overall risk of bias (per your assessment):
   ☐ Low
   ☐ Moderate
   ☐ High

 © 2026. The Author(s).

6.3 Reporting guideline mentioned by authors:
☐ TRIPOD/TRIPOD–AI
☐ STARD/STARD–AI
☐ CONSORT/SPIRIT
☐ None reported

7. Transparency, calibration, and clinical utility
7.1 Code availability:
☐ Publicly available (provide link if given): __________
☐ Available on request
☐ Not available/not reported

7.2 Data availability:
☐ Public dataset used (name): __________
☐ Study data shared publicly (link): __________
☐ Available on request
☐ Not available/not reported

7.3 Calibration analysis reported?
☐ Yes – specify (e.g., calibration plot, Brier score): __________
☐ No

7.4 Clinical utility analysis reported?
☐ Yes – specify (e.g., decision–curve analysis, net benefit): __________
☐ No

8. Key conclusions and reviewer comments
8.1 Authors' main conclusions (1–3 sentences):
8.2 Reviewer's notes/concerns (e.g., significant limitations, applicability):

Supplementary Table S6 Eligibility criteria

Study framework	Inclusion criteria	Exclusion criteria
Study type	• AI-related technology in dentistry: Reported on the methodologies and applications of artificial intelligence (AI) or machine learning (ML) in dentistry<br>• Technology advances to improve the quality of resin-based composites as restorative materials	Studies not applied to dentistry<br>Other restorative materials<br>Other digital technologies, including CAD/CAM and 3D printing
Intervention	• Artificial intelligence<br>• Machine learning<br>• Deep learning	Not AI-related technology and Robotic dentistry
Comparator	• Other composite types<br>• Other technologies<br>• Any comparator	No comparator
Outcomes	• Evaluations of the mechanical quality of dental composites: *strength, hardness, flexural strength, fracture toughness, wear resistance, stiffness (Modulus of Elasticity) & polymerisation quality*<br>• Evaluation of aesthetic quality: *Colour matching, translucency, polishability, fluorescence, & Opalescence*<br>Evaluation of biocompatibility	Not directly related to the mechanical or aesthetic quality or biocompatibility of composite resins
Study Design	Experimental, in vivo, in vitro	Virtual or modelling studies, Letters to the editor, commentaries, Narrative reviews, systematic reviews, conference abstracts, dissertations, and theses
Filters to apply	Limit to 2020–2025	Before 2020
Databases to search (4 in total)	PubMed, Embase, Scopus, Web of Science	

 © 2026. The Author(s).

Supplementary Table S7 Dataset characteristics and validation approaches for included AI studies on composite restorations

Study (first author, year)	Sample type/domain	Dataset size (*n*)	Class balance (if applicable)	Validation strategy	External/multicentre data
Almoro 2024	Intraoral images, shade matching	1,253 images	Not reported	Train–test split plus cross-validation; 30% of training set for test, separate 20-image user test set	Single-centre (Philippines)
Dilian and Kadhim 2022	Extracted premolars, microleakage	60 teeth (84 observations)	Not applicable	Not reported	Single-centre (Iraq)
Engels 2022	Intraoral photos, restoration classification	1,761 images	483 unrestored, 570 composite, 213 cement, 278 amalgam, 125 gold, 92 ceramic	Single train–test split (20% test)	Single-centre (Germany)
Karatas 2021	Bitewing & periapical radiographs	500 images (275 bitewings, 225 periapical)	Amalgam, composite, metal-ceramic (exact counts not given)	5-fold cross-validation plus single 20% hold-out test	Single-centre (Turkey)
Paniagua 2025	Commercial composite specimens, performance prediction	233 samples	Not applicable (continuous outcomes)	Single train–test split (20% test)	Single-centre (USA)
Rocha 2022	Resin-based composites (conventional + bulk fill), cure depth	150 specimens	Not applicable	Not reported (ANN evaluated via ASE)	Multinational (USA, Canada, Brazil), but no external test set is described
Shubham and Banerjee 2024	Intraoral photos, restoration classification	10,220 images	3,360 amalgam; 3,450 composite; 3,410 metal-ceramic	Single train–test split (test proportion not reported)	Single-centre (India)
Suryawanshi and Behera 2023a	In vitro composite specimens, FS & VH	12 data points	Not applicable	Single train–test split (40% test)	Single-centre (India)
Suryawanshi and Behera 2023b	In vitro composite specimens, abrasive wear	72 specimens	Not applicable	Single train–test split (15% test)	Single-centre (India)
Suryawanshi and Behera 2024	In vitro composite specimens, wear	72 specimens	Not applicable	Single train–test split (20% test)	Single-centre (India)
Takahashi 2021	Intraoral images (arches), prosthesis/restoration detection	1,904 images	Includes composites and other materials (exact distribution not reported)	Single train–test split (20% test)	Single-centre (Japan)
Tejada–Casado 2022	Disk-shaped layered resin composites, color prediction	25 enamel–dentine combinations	Not applicable	Single train–test configuration; 5-sample training set (20 samples) and 9-sample training set (16 samples)	Multicenter team (Spain/Romania), but no external dataset
Varshney 2024	Restorative materials in paediatric dentistry, color change	200 specimens	GIC, RMGIC, Microhybrid Nanohybrid composites (distribution not specified)	Single train–test split (30% test)	Single-centre (India)
Wang 2023	Commercial dental adhesives, μTBS prediction	180 μTBS values from 81 adhesives	Not applicable (binary/ordinal strength categories)	10-fold cross-validation with nested CV	Single-centre (USA)

 © 2026. The Author(s).

Supplementary Table S8 Reporting, transparency, and clinical utility characteristics of included studies

Study (first author, year)	Study type (prediction/diagnostic/in vitro experimental)	Reporting guideline mentioned (TRIPOD/TRIPOD–AI/STARD/STARD–AI/none)	Code publicly available (yes/no/partial)	Data publicly available (yes/no/derived from public dataset)	Calibration analysis reported (yes/no; type)	Clinical utility analysis (e.g., decision–curve) (yes/no; type)
Almoro 2024	Diagnostic imaging (shade matching)	None reported	No	Partially public (uses 1,136 images from an online labelled dataset plus 117 locally labelled images; full combined dataset not shared)	No	No
Engels 2022	Diagnostic imaging (restoration classification)	STARD	Partial<br>Web app only (no source code)	No (clinical images not shared)	Yes (Rater calibration with kappa)	No (clinical implications discussed narratively)
Karatas 2021	Diagnostic imaging (radiographs)	None reported	No	No	Yes<br>(ROC & AUC reported per restoration type)	No formal analysis (no decision–curve or similar)
Shubham and Banerjee 2024	Diagnostic imaging/prediction (AI classification of restorations from images)	None reported	No	No (10,220-image dataset used, but not stated as publicly available)	Yes – discrimination metrics & ROC reported (accuracy, precision, recall, F1, confusion matrices, ROC)	No formal analysis (no decision–curve; clinical implications discussed narratively)
Study (first author, year)	Study type (prediction/diagnostic/in vitro experimental)	Reporting guideline mentioned (TRIPOD/TRIPOD–AI/STARD/STARD–AI/none)	Code publicly available (yes/no/partial)	Data publicly available (yes/no/derived from public dataset)	Calibration analysis reported (yes/no; type)	Clinical utility analysis (e.g., decision–curve) (yes/no; type)
Takahashi 2021	Diagnostic imaging (object detection)	None reported	No	No<br>Available on request only (not publicly available due to privacy/ethics)	No (AP/mAP/mIoU only))	No formal analysis (no decision–curve; technical performance only)
Paniagua 2025	Prediction model (materials)	None reported	No	Partial<br>Derived from published studies (curated from >200 publications; no standalone dataset shared)	No (accuracy/precision/recall/F1/AUC only)	No (focus on material development, not direct clinical decisions)
Rocha 2022	Prediction model *in vitro* experimental (cure depth)	None reported	Yes<br>ANN algorithm/code available supplementary materials	No (Results and plots in supplementary only)	Yes<br>calibrated instruments and ANN error minimisation	No formal analysis (no decision–curve; clinical implications discussed narratively)
Suryawanshi and Behera 2023a	Prediction model *in vitro* experimental (flexural strength/Vickers micro-hardness)	None reported	No (developed in Python, but no public code statement)	No (experimental data only presented as tables/figures	No (only internal performance metrics reported)	No (focus is on mechanical prediction, not clinical decisions)

 © 2026. The Author(s).

Supplementary Table S8 (*Continued*)

Study (first author, year)	Study type (prediction/ diagnostic/in vitro experimental)	Reporting guideline mentioned (TRIPOD/ TRIPOD–AI/STARD/ STARD–AI/none)	Code publicly available (yes/no/partial)	Data publicly available (yes/no/derived from public dataset)	Calibration analysis reported (yes/no; type)	Clinical utility analysis (e. g., decision–curve) (yes/ no; type)
Suryawanshi and Behera 2023b	Prediction model *in vitro* experimental (abrasive wear)	None reported	No (ANN implemented, but no statement of publicly available code)	No (experimental &predicted wear data reported only within the article, not as a separate public dataset)	No (only internal performance metrics reported)	No (focus is on predicting wear behavior, not clinical decision–making)
Suryawanshi and Behera 2024	Prediction model *in vitro* experimental (wear)	None reported	No (ML models described, but no public code statement.)	No (*in vitro* experimental data generated by the authors)	No (only internal performance metrics reported)	No (no decision–curve or clinical impact analysis reported.)
Tejada–Casado 2022	Prediction model *In vitro* experimental Unsupervised/color prediction	None reported	No (PCA-based algorithm described, but no statement that code is publicly available)	No (spectral reflectance data generated in vitro; not stated as a public dataset)	Yes (calibrated spectroradiometer RMSE and GFC used as performance measures)	No (no formal analysis – PT/AT CIEDE2000 thresholds used for dental relevance, but no decision–curve or formal clinical impact analysis)
Study (first author, year)	Study type (prediction/ diagnostic/in vitro experimental)	Reporting guideline mentioned (TRIPOD/ TRIPOD–AI/STARD/ STARD–AI/none)	Code publicly available (yes/no/partial)	Data publicly available (yes/no/derived from public dataset)	Calibration analysis reported (yes/no; type)	Clinical utility analysis (e. g., decision–curve) (yes/ no; type)
Varshney 2024	*In vitro* experimental Color prediction	None reported	No (CHAID and CART ML methods described, but no statement that code is publicly available)	Yes article data available under CC BY 4.0/public domain terms	Yes (digital spectrophotometer calibrated against a white reference for color measurements)	No (no decision–curve or other formal clinical utility analysis reported)
Wang 2023	Prediction model (microtensile bond strength: μTBS)	None reported	No (ML code not stated as publicly available)	No Derived from public sources (μTBS and composition from literature/brochures; no separate shared dataset).	No (performance assessed with AUC, accuracy, stratified and nested cross-validation, but no explicit calibration analysis)	No (discusses potential to aid material development & performance prediction, but no formal clinical utility or decision–curve analysis)
Dilian & Kadhim 2022	*In vitro* experimental (marginal microleakage comparison in composite resins)	None reported	No (random forest and other analyses described, but no statement of publicly available code)	No (study data not stated as publicly available; randomization with a random number generator & analysis in R 4.1.3 only)	Yes (random forest performance reported (accuracy, pseudo $R^2 = 0.671$) with no dedicated calibration plots or statistics)	No (no decision–curve or other formal clinical utility analysis reported)

Abbreviations: AP, average precision; AUC, Area under the curve; mAP, mean average precision; CART, Classification And Regression Trees; CHAID, Chi-squared Automatic Interaction Detection; GFC, Goodness of Fit Coefficient; mIoU, mean intersection over union; ML, Machine learning; PT, perceptibility threshold; RMSE, Root Mean Square Error; ROS, Receiver operating characteristics; STARD, Standard for Reporting of Diagnostic Accuracy Studies; TRIPOD, TRansparent Reporting of a multivariable Individual Prognosis Or Diagnosis prediction model.

 © 2026. The Author(s).

Supplementary Table S9 Risk of bias in included studies based on QUIN assessment instrument

Criteria	Dilian & Kadhim, 2022	Rocha et al, 2022	Suryawanshi & Behera, 2024	Suryawanshi & Behera, 2023a	Suryawanshi & Behera, 2023b	Tejada-Casado et al, 2022	Varshney et al, 2024
Clearly stated aims/objectives	2	2	2	2	2	2	2
Detailed explanation of sample size calculation	0	2	0	0	0	0	2
Detailed explanation of the sampling technique	1	2	1	1	1	2	1
Details of the comparison group	2	2	2	2	2	2	2
Detailed explanation of methodology	2	2	2	2	2	2	2
Operator details	0	2	1	1	2	2	1
Randomization	2	2	0	0	0	0	0
Method of measurement of outcome	1	2	2	2	2	2	2
Outcome assessor details	2	2	0	0	0	0	2
Blinding	1	2	0	0	0	1	0
Statistical analysis	2	2	2	2	2	2	2
Presentation of results	2	2	2	2	2	2	2
Overall score	17	24	14	14	15	18	18
Risk of bias	M	L	M	M	M	L	L

Abbreviations: H, High; L, Low; M, Moderate.

Supplementary Table S10 Risk of bias in included studies based on PROBAST assessment instrument

Criteria	Risk of bias				Applicability			Overall prediction	
	Participants	Predictors	Outcome	Analysis	Participants	Predictors	Outcome	Risk of bias	Applicability
Paniagua et al, 2025	+	?	?	?	+	+	?	?	?
Shubham & Banerjee, 2024	+	+	+	+	+	+	+	+	+
Wang et al, 2023	+	+	+	+	+	+	+	+	+

Abbreviation: PROBAST, Prediction model of Risk of Bias Assessment Tool.
+ low risk of bias or low concern regarding applicability; – high risk of bias or great concern regarding applicability.
? unclear risk of bias or risk of concern regarding applicability.

 © 2026. The Author(s).

Key to using the PROBAST assessment tool

Domains
1. Participants
1. Were appropriate data sources used, eg, cohort, RCT, or nested case-control study data?
2. Were all the inclusions and exclusions of participants appropriate?
2. Predictors
1. Were predictors defined and assessed in a similar way for all participants?
2. Were predictor assessments made without knowledge of outcome data?
3. Are all predictors available at the time the model is intended to be used?
3. Outcome
1. Was the outcome determined appropriately?
2. Was a prespecified or standard outcome definition used?
3. Were predictors excluded from the outcome definition?
4. Was the outcome defined and determined in a similar way for all participants?
5. Was the outcome determined without knowledge of predictor information?
6. Was the time interval between predictor assessment and outcome determination appropriate?
4. Analysis
1. Were there a reasonable number of participants with the outcome?
2. Were continuous and categorical predictors handled appropriately?
3. Were all enrolled participants included in the analysis?
4. Were participants with missing data handled appropriately?
5. Was the selection of predictors based on univariable analysis avoided?
6. Were complexities in the data (eg, censoring, competing risks, and sampling of control participants) accounted for appropriately?
7. Were relevant model performance measures evaluated appropriately?
8. Were model overfitting and optimism in model performance accounted for?
9. Do predictors and their assigned weights in the final model correspond to the results from the reported multivariable analysis?

Supplementary Table S11 Risk of bias in included studies based on QUADAS-2 assessment instrument

Study	Patient selection					Index test				Reference standard				Flow and timing					Overall
	1	2	3	4	5	6	7	8	9	10	11	12	13	14	15	16	17	18	
Almoro et al, 2024	U	Y	U	H	H	U	Y	H	H	Y	U	H	L	U	U	U	U	H	HIGH
Engels et al, 2022	U	Y	Y	L	L	Y	U	L	L	Y	Y	L	L	Y	Y	Y	Y	L	LOW
Karatas et al, 2021	U	Y	U	H	H	Y	U	U	L	Y	Y	L	L	Y	Y	Y	U	H	MODERATE
Takahashi et al, 2021	U	Y	U	H	L	Y	Y	U	L	Y	Y	L	L	Y	Y	Y	Y	L	MODERATE

Abbreviations: H, High; L, Low; N, No; U, Unclear; Y, Yes.

 © 2026. The Author(s).

Patient Selection

1. Was a consecutive or random sample of patients enrolled? (Yes/No/Unclear)
2. Was a case–control design avoided? (Yes/No/Unclear)
3. Did the study avoid inappropriate exclusions? (Yes/No/Unclear)
4. Could the selection of patients have introduced bias? (RISK: LOW/HIGH/UNCLEAR)
5. Is there concern that the included patients do not match the review question? (RISK: LOW/HIGH/UNCLEAR)

Index Test

6. Were the index test results interpreted without knowledge of the results of the reference standard? (Yes/No/Unclear)
7. If a threshold was used, was it pre-specified? (Yes/No/Unclear)
8. Could the conduct or interpretation of the index test have introduced bias? (Low/High/Unclear)
9. Is there concern that the index test, its conduct, or interpretation differs from the review question? (RISK: LOW/HIGH/UNCLEAR)

Reference Standard

10. Is the reference standard likely to correctly classify the target condition? (Yes/No/Unclear)
11. Were the reference standard results interpreted without knowledge of the results of the index test? (Yes/No/Unclear)
12. Could the reference standard, its conduct, or its interpretation have introduced bias? (RISK: LOW/HIGH/UNCLEAR)
13. Is there concern that the target condition, as defined by the reference standard, does not match the review question? (RISK: LOW/HIGH/UNCLEAR)
14. Was there an appropriate interval between index test(s) and reference standard? Yes/No/Unclear
15. Did all patients receive a reference standard? (Yes/No/Unclear)
16. Did patients receive the same reference standard? (Yes/No/Unclear)
17. Were all patients included in the analysis? (Yes/No/Unclear)
18. Could the patient flow have introduced bias? (RISK: LOW/HIGH/UNCLEAR)

 © 2026. The Author(s).
